# Improving the quality of the Global Burden of Disease tuberculosis estimates from the Institute for Health Metrics and Evaluation

**DOI:** 10.1093/ije/dyad128

**Published:** 2023-09-27

**Authors:** Peter J Dodd, Christopher Finn McQuaid, Prasada Rao, Ibrahim Abubakar, Nimalan Arinaminpathy, Anna Carnegie, Frank Cobelens, David Dowdy, Kathy Fiekert, Alison D Grant, Jing Wu, Faith Nekabari Nfii, Nabila Shaikh, Rein M G J Houben, Richard G White

**Affiliations:** Sheffield Centre for Health and Related Research, University of Sheffield, Sheffield, UK; TB Modelling Group, TB Centre and Centre for Mathematical Modelling of Infectious Diseases, London School of Hygiene & Tropical Medicine, London, UK; Former Health Secretary, Government of India, Bangalore, India; University College London, London, UK; MRC Centre for Global Infectious Disease Analysis; and the Abdul Latif Jameel Institute for Disease and Emergency Analytics, School of Public Health, Imperial College London, London, UK; Centre for Mathematical Modelling of Infectious Diseases, London School of Hygiene and Tropical Medicine, London, UK; Department of Global Health and Amsterdam Institute for Global Health and Development, Amsterdam University Medical Centers location University of Amsterdam, Amsterdam, Netherlands; Johns Hopkins Bloomberg School of Public Health, Baltimore, USA; KNCV Tuberculosis Foundation, The Hague, Netherlands; TB Centre, London School of Hygiene & Tropical Medicine, London, UK; Africa Health Research Institute, School of Laboratory Medicine & Medical Sciences, College of Health Sciences, University of KwaZulu-Natal, Durban, South Africa; Center for Chronic Diseases Prevention and Control, China CDC, Beijing, China; Africa Union-Africa Centres for Disease Control and Prevention, Addis Ababa, Ethiopia; TB Modelling Group, TB Centre and Centre for Mathematical Modelling of Infectious Diseases, London School of Hygiene & Tropical Medicine, London, UK; Influenza & COVID-19 Franchise, Sanofi, Reading, UK; TB Modelling Group, TB Centre and Centre for Mathematical Modelling of Infectious Diseases, London School of Hygiene & Tropical Medicine, London, UK; TB Modelling Group, TB Centre and Centre for Mathematical Modelling of Infectious Diseases, London School of Hygiene & Tropical Medicine, London, UK

**Keywords:** Tuberculosis, Global Burden of Disease, Institute for Health Metrics and Evaluation, World Health Organization, policy

## Background

Tuberculosis (TB) remains a leading infectious cause of death worldwide.^1^^,^^2^ But how do we know this? Two organizations publish annual estimates of the global and national burden of TB disease: the World Health Organization (WHO) and the Institute for Health Metrics and Evaluation (IHME).

TB decision-makers are fortunate to have two organizations producing annual burden estimates, but there are differences between the estimates from these two organizations. Differences between the estimates can be considered as useful signs of genuine uncertainty, but may also confuse decision-makers.

Over time, both organizations have sought to understand the reasons for these differences, improved their methods and called for the strengthening of the data collection systems on which these estimates rely. In 2015 a comparison of the WHO’s and IHME’s methods and results was carried out by WHO staff^3^ and in 2018 an independent group explored differences between estimates of TB mortality.^4^ Outside of TB, other reviews of IHME methods have been carried out, including the methods used to estimate the disease burden due to undernutrition and suboptimal breastfeeding.^5^

Another such initiative, focusing on IHME’s TB estimates, was convened by the Independent Advisory Committee (IAC) for the Global Burden of Disease (GBD). The IAC’s remit is wide-ranging and includes commissioning ‘Deep Dives’ into specific topics considered of high importance.^4^ The first of these Deep Dives in 2020 was into the methods used to estimate the ‘Local Burden of Disease’.^6^

In the second of these Deep Dives, the IAC commissioned a review of IHME’s TB estimates and to make actionable recommendations to improve its quality and usefulness. This article reports on the quantitative work identifying recommendations to improve its quality. A second article describing a qualitative analysis of stakeholder interviews to inform its usefulness has been written up separately.

## Key recommendations

IHME’s GBD should:

- Place greater emphasis on clear, self-contained explanations of methods and more meaningful reproducibility, more similar to WHO standards
- strengthen comparisons and dialogue with WHO to systematically identify and understand differences in data and methods
- consider refining methods to enable stronger links between TB estimates and country data, and to identify and explain issues, such as disease notifications exceeding disease incidence
- consider updating or justify the rationale for equal duration of disease by HIV and sex, which appears inconsistent with empirical data.

## Our investigation

A consultant Panel, supported by a group of subject matter experts, agreed on a set of questions to explore within the scope of the exercise. The Panel and Expert Group membership is listed in an appendix and included epidemiologists, public health specialists, statisticians, public health leaders and programmatic experts. The Terms of Reference for this evaluation defining our questions and approach were drawn up over 4 months by the Global Burden of Disease Independent Advisory Committee Panel, with input from subject matter experts and IHME researchers. The Panel worked with IHME’s GBD TB Team, to experiment with input data/analysis decisions to better understand the impact on the final estimates by any changes/tweaks made to the input data (not reported here). Experimentation, along with the other parts of the review, helped to identify and prioritize areas in which improvements to the TB estimates could be made. The Panel also sought to understand the impact of some of the less-well-understood elements of the estimation methods on TB burden. The process took place over 9 months and involved monthly meetings of the Expert Group to give input and direction, and data collation, analysis and review with the Panel and the Project Support team. We focused on understanding data sources, how data are processed and used, documentation review and exploration of generated outputs. Particular areas of focus were informed by previous work comparing WHO and IHME TB estimates and suggestions from an Expert Group and the IHME TB team. Priority was given to areas that might inform specific actionable recommendations. The IHME TB team provided additional data from their analysis processes, were able to perform some suggested experiments and helped with explanations and additional documentation.

In this article, we present only selected analyses reproducible with publicly available data, focusing on 30 high-TB-burden countries accounting for ∼80% of global TB incidence.

Data analyses included visual comparisons between empirical data, IHME estimates and WHO estimates. Age- and sex-disaggregated incidence estimates and TB notification data were graphed. To explore sex differences in rates of TB detection, prevalence-to-notification (P:N) ratios, stratified by sex, were constructed for countries with TB prevalence surveys using TB notification data from the same year and compared with P:N ratios based on IHME TB prevalence estimates. To explore the impact of HIV infection on the mean duration of TB disease, we also examined HIV-stratified ratios of IHME TB prevalence-to-incidence estimates. We compared additional aspects of IHME and WHO estimates, including the relative uncertainty in incidence estimates (defined as uncertainty interval widths as a proportion of central estimates), incidence estimate trends over time and ratios of mortality-to-incidence estimates by age and sex.

Additional plots to support points in the text are contained in supplementary material. The code and data for these analyses are publicly available on GitHub.

## Observations and recommendations

Below we present our grouped observations together with associated evidence and recommendations.

**Figure 1.**
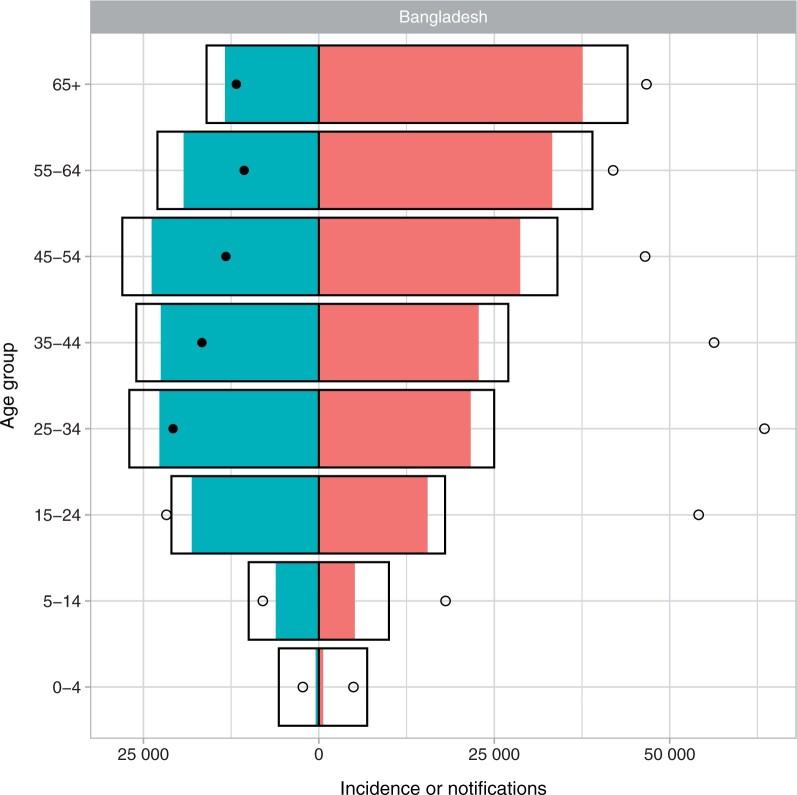
New and relapse tuberculosis notifications and estimated tuberculosis incidence by age and sex. Coloured bars are notifications; open bars are World Health Organization (WHO) incidence estimates; circles are Institute for Health Metrics and Evaluation (IHME) incidence estimates. Filled circles suggest a case-detection ratio greater than one according to these estimates. Men to the right; women to the left. Bangladesh has been chosen as an example country; see Supplementary File (available as Supplementary data at *IJE* online) for 30 high-TB-burden countries

**Figure 2.**
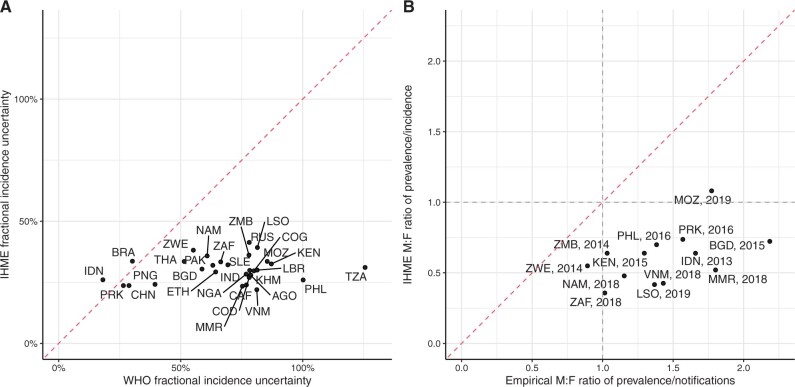
Relative uncertainty in tuberculosis incidence estimates and male-to-female ratios of empirical tuberculosis prevalence:notification ratios. (A) Relative uncertainty* in 2019 tuberculosis incidence estimates from the Institute for Health Metrics and Evaluation (IHME) vs the World Health Organization (WHO). (B) Male-to-female ratio of empirical tuberculosis prevalence:notification ratio vs male-to-female ratio of IHME estimated prevalence:incidence ratio. ^†^The red lines show equality. *Defined as the uncertainty range divided by the midpoint estimate. ^†^A ratio of <1 suggests that females have a higher prevalence:notification or prevalence:incidence ratio, whereas a ratio of >1 suggests that males have a higher ratio. AGO, Angola; BGD, Bangladesh; BRA, Brazil; KHM, Cambodia; CAF, Central African Republic; CHN, China; COG, Congo; PRK, Democratic People’s Republic of Korea; COD, Democratic Republic of the Congo; ETH, Ethiopia; IND, India; IDN, Indonesia; KEN, Kenya; LSO, Lesotho; LBR, Liberia; MOZ, Mozambique; MMR, Myanmar; NAM, Namibia; NGA, Nigeria; PAK, Pakistan; PNG, Papua New Guinea; PHL, Philippines; RUS, Russian Federation; SLE, Sierra Leone; ZAF, South Africa; THA, Thailand; TZA, United Republic of Tanzania; VNM, Viet Nam; ZMB, Zambia; ZWE, Zimbabwe

- **Methodological explanations are hard to follow and reproducibility is limited.** Despite intensive efforts over many years, many Panel and Expert Group members struggled to understand IHME documentation. Current methodological appendices to publications are not always self-contained or intelligible to qualified readers from outside IHME. It would be useful to publish a clear academic article on IHME TB estimation methods aimed at readers with quantitative experience. In order to convey the dependence of output estimates on input data and processing steps, it would be useful to develop simplified process charts that include quantitative information on percentage changes at each step, e.g. relative changes in TB deaths from vital registration, through ‘CoD correct’ and each other step through to final estimates. Although the IHME codebase is public, more effort should be made to identify shareable sub-analyses specific to TB estimates that include data and code and are genuinely reproducible by others. In addition, these descriptions should include key assumptions to support an understanding of how data and models interact. For example, issues raised in Items (iv) and (v) below highlight specific choices that are made at key steps, which are not currently clear in the methods of reporting.
  We recommend greater emphasis on clear, self-contained explanations of methods and on more meaningful reproducibility.
- **Differences between** IHME **and WHO estimates should be monitored and understood.** Discrepancies between IHME and WHO estimates are to be expected and are often useful signatures of genuine uncertainty. IHME and WHO incidence and mortality estimates did have notable differences in trends for some countries with large burdens, including India, Nigeria, South Africa and Bangladesh (see Supplementary Figures S1 and S2, available as Supplementary data at *IJE* online, for phase portraits of GBD vs WHO estimates for 2010–19). Percentage differences in 2019 ranged from 0% to 75% for estimated TB incidence and from 4% to 88% for TB mortality. TB/HIV estimates typically differed between IHME and WHO by a larger amount than all-TB estimates (Supplementary Figure S3, available as Supplementary data at *IJE* online). IHME incidence estimates were less strongly informed by notification data than WHO estimates and more commonly implied substantial overdiagnosis for some years or age groups than WHO estimates, i.e. estimated a case-detection ratio of >1 (e.g. see Bangladesh in Figure 1 and Supplementary Figure S4, available as Supplementary data at *IJE* online, for other countries). The relative uncertainty in IHME incidence estimates was smaller than in WHO estimates and less variable across our 30 focus countries (Figure 2a).
  We recommend strengthening comparisons and dialogue with WHO to systematically identify and understand differences in data and methods, particularly for high-burden countries with divergent trends.
- **Relationships with country data can be hard to understand and interpret**. Because of the indirect way in which much data influence IHME estimates in a given country (often estimates are affected by data in other countries), it can be difficult to see how particular pieces of country data influence estimates. For example, it can be difficult to see how changes in data in a particular country (e.g. TB notifications) lead to changes in its estimates or to changes in the consistency between data and estimates. Some relationships with data, such as incidence estimates that are lower than notifications in a given year, in total or in particular age/sex groups (see Figure 1), would have important programmatic implications if true. Although notifications can exceed incidence due to false positive clinical diagnoses, inconsistencies have the potential to undermine country trust in either IHME estimates or in surveillance systems and, if false, could motivate potentially wasteful or even harmful programmatic interventions.
  Further, the relationship between prevalence estimates by age/sex and prevalence survey data from the same year varied. For some countries, all-TB prevalence was similar to bacteriologically confirmed prevalence from the survey across age groups, whereas for others it was larger or smaller (see Supplementary Figure S5, available as Supplementary data at *IJE* online).
  We recommend consideration of method changes that allow TB estimates in a country to be more strongly influenced by that country’s data (as opposed to data in other countries) and reporting or visualization approaches that permit routine identification and explanation of important discrepancies between country estimates and country data.
- **Some epidemiological patterns are not consistent with external information/expectation.** Prior to widespread antiretroviral therapy (ART) for people living with HIV (PLHIV), evidence and anecdote suggested a much shorter typical duration of TB disease among PLHIV.^7^^,^^8^ More recent analyses of P:N ratios stratified by HIV still suggest a shorter duration of TB disease among PLHIV.^9^ The IHME estimates imply a duration of TB that does not differ by HIV status, which is at odds with this expectation (see Supplementary Figure S6, available as Supplementary data at *IJE* online, for IHME TB prevalence/incidence stratified by HIV status). Similarly, P:N analyses suggest longer typical durations of TB disease among men in almost all high-burden countries.^9^^,^^10^ IHME estimates however implied durations slightly higher for women across most ages in most settings (see Figure 2b). Finally, it would generally be expected that as the implied case-detection ratio increased, the implied average case-fatality ratio would decrease reflecting better outcomes for people with TB, of whom an increasing proportion would have received TB treatment. However, the IHME estimates typically assumed that the case-fatality ratio remained relatively unchanged despite changes in the assumed case-detection ratio (see Supplementary Figure S7, available as Supplementary data at *IJE* online, for a phase portrait of the TB case-fatality ratio vs the case-detection ratio for 2010–19).
  We recommend systematically and routinely outputting diagnostic metrics such as these for estimates and greater use, or weighting, of sex- and HIV-stratified data.
- **An unexpected stability across settings, over time and in relation to change.** A number of patterns in the estimates showed a lack of variation that was highlighted as surprising, potentially indicating a lack of response to (local) data or an artefact of model formulation. For example, the patterns of the implied duration of disease were similar by age and sex across most settings, even when varying in absolute level (see Supplementary Figure S8, available as Supplementary data at *IJE* online, for IHME TB prevalence/incidence by age and sex). The implied duration of disease was typically very stable over time in each country (except the Philippines), whereas one would expect a decline with improving TB detection and management of HIV (see Supplementary Figure S9, available as Supplementary data at *IJE* online, for IHME TB prevalence/incidence by year and sex). The relative uncertainty in incidence estimates varied surprisingly little between countries given the expected differences in data availability and quality (see Figure 2a).
  We recommend exploration of the degree of statistical smoothing inherent in the estimates (between countries, age groups and other stratifications) and/or the use of covariates that can capture changes in TB programmes (e.g. rates of TB assessment of bacteriological confirmation).
- **Use of projection estimates**. To make projections for the future burden of disease, IHME uses estimates from 2016 to predict the burden for the years 2017–40 modelling cause-specific mortality based on (a) risk factors and a limited number of interventions (for HIV, family planning and routine vaccination), (b) socio-demographic projections and (c) unexplained variation and the past rates of change in these components. The use of a forecasting methodology that did not consider TB interventions suggests that these projection estimates should be used with caution for TB.
  We recommended that IHME reflect on the purposes and consequences of future TB projection estimates, given that they do not take into account programmatic changes for TB. If the intent is to support decision-making, TB could be modelled explicitly including potential major future programmatic changes.

## Discussion

During the review, we identified recommendations to improve the quality of IHME’s TB estimates. Our key recommendations covered strengthening explanations of methods and reproducibility, strengthening comparisons and dialogue with WHO, enabling stronger links between estimates and country data including explaining likely problematic differences, justifying or updating the equal duration of disease by HIV and sex, re-evaluating statistical smoothing and reflecting on the utility of TB projections.

All estimates have shortcomings that may limit their appropriateness for particular uses. For example, use of estimates for target-setting requires particular caution, especially when considering subpopulations, where estimates have higher proportional uncertainty. Since GBD TB estimates are more weakly informed by TB notifications, their use in understanding trends in case finding may not be appropriate. Similarly, not including the effects of programme changes in projections limits their usefulness in forecasting future scenarios. Users should exercise judgement in applying these and other estimates for their purposes.

Not all aspects of the IHME estimates that have been highlighted are necessarily problematic. However, for most of these potentially problematic aspects (e.g. TB notifications exceeding incidence estimates), there is no obvious pattern in data availability or quality that could explain their occurrence. These aspects may reflect differences in philosophy and design between the IHME and WHO estimation processes, in particular in relation to the reliability of TB notification data, and the degree to which estimates in a country are influenced by data from other countries.

The approach taken by WHO is to work with country TB programmes to collate notification and other surveillance data, and invite countries to comment on draft estimates. As part of this, there is strong attention on the relationship between incidence estimates and the notification data that TB programmes are familiar with and are responsible for. Part of WHO’s remit is to encourage and assist in improvements in TB surveillance systems. Estimates that lack a visible relation with or response to notification data could harm these efforts. While acknowledging the problems with notification data, notifications are central to WHO estimates of incidence. For countries that lack vital registration data (notably in sub-Saharan Africa), WHO estimates of TB mortality are derived from incidence estimates by applying case-fatality ratios from literature. Therefore, WHO estimates of incidence and mortality for a given country are based on data from that country (together with explicit assumptions based on literature).

IHME appears to regard TB notification data as less reliable than data on deaths and estimates of TB incidence are mostly derived from estimates of TB mortality (with input from TB prevalence and notification data as covariates). Data on TB deaths are usually less available to TB programmes and, in a substantial number of high-TB-burden countries, do not exist at all. This means the relationship between incidence and familiar programme data is less clear and where data on deaths are lacking, predicted deaths and case-fatality ratios are most influenced by data from settings with vital registration systems. The implicit reliance on data from other countries in this approach greatly complicates the understanding of the methods and the drivers of the estimates, and lessens the responsiveness of local estimates to changes in local data. Different choices of statistical model structure, hyperparameters or covariates may allow local responsiveness to be increased.

One aspect of the IHME TB estimation process that may be relatively accessible to change, and was associated with two queries around face validity, is the way evidence on TB disease duration is used. This is important as it determines the relationship between incidence and prevalence. Currently, duration appears not to depend on HIV status or sex, but the Expert Group and external data^9^^,^^10^ strongly suggest that it should. Including dependencies on HIV and/or sex should be possible either by use of literature data if judged sufficiently applicable or by modelling of stratified notification and prevalence data. It may also be worth considering how duration is likely to change over time and in response to programme changes, and what evidence would support alternative approaches to the current dependence on a single generic healthcare quality index. In turn, those decisions and rationale could/should be included in the next update of IHME estimates for TB.

Another general theme in recommendations is around communications/interaction with other stakeholders. In particular, dialogue with country TB programmes would provide a useful source of feedback, scrutiny and help to understand the local plausibility and implications of predictions, as well as build trust and encourage the use of estimates. This was a strong theme emerging from an ongoing qualitative analysis (pc Anna Carnegie. ‘Estimation above engagement? Estimates of TB burden and their utility in policy decision-making’). Ongoing dialogue with WHO would help build a mutual understanding of methods and data, as well as identifying areas in which WHO could advocate for and support additional or improved data collection. The two bodies could consider co-developing validation and comparison checklists to flag results for additional attention. More sustained traditional engagement with the global TB epidemiology academic research community would enable an improved understanding of methods and facilitate feedback. The current model of the collaborator network seems more focused on data acquisition in return for co-authorship. Finally, developing tools that allow end users to understand the relationships between input data (including flagging its absence) and final estimates would be valuable.

While it is clear that IHME and WHO estimates have great value in their complementarity, our independent evaluation identified areas for improvement. Other disease areas will likely benefit from a similar assessment to increase the quality of the burden estimates and projections provided by IHME.

## Ethics approval

Ethics approval was not required for this study, which used publicly available, fully anonymized data.

## Supplementary Material

dyad128_Supplementary_Data

## Data Availability

The code and data for these analyses are publicly available at https://github.com/petedodd/ihmexplore.
