# Supplementary material for: Improving the quality of the Global Burden of Disease tuberculosis estimates from the Institute for Health Metrics and Evaluation
Source: Int J Epidemiol. 2023 Sep 27;52(6):1681–6. doi: 10.1093/ije/dyad128 (PMC10749761; doi:10.1093/ije/dyad128)
Supplement: dyad128_Supplementary_Data [file dyad128_supplementary_data.pdf]

Improving the quality of the Institute for Health Metrics and Evaluation's Global Burden of Disease tuberculosis estimates: Supplementary Figures

Peter J. Dodd[1], C. Finn McQuaid[2], Prasada Rao[3], Ibrahim Abubakar[4], Nim Arinaminpathy[5], Anna Carnegie[2], Frank Cobelens[6], David Dowdy[7], Kathy Fiekert[8], Alison Grant[9], Jing Wu[10], Faith Nekabari Nfii[11], Nabila Shaikh[12], Rein M G J Houben[2], Richard White[2]

[1]School of Health and Related Research, University of Sheffield, Sheffield, UK
[2]TB Modelling Group, TB Centre, London School of Hygiene &Tropical Medicine, London, UK
[3]Former Health Secretary, Government of India
[4]University College London, UK
[5]MRC Centre for Global Infectious Disease Analysis; and the Abdul Latif Jameel Institute for Disease and Emergency Analytics, School of Public Health, Imperial College London
[6]The Amsterdam Institute for Global Health and Development, Netherlands
[7]Johns Hopkins Bloomberg School of Public Health, USA
[8]KNCV Tuberculosis Foundation, Netherlands
[9]TB Centre, London School of Hygiene &Tropical Medicine, London, UK; Africa Health Research Institute, South Africa
[10]Center for Chronic Diseases Prevention and Control, China CDC, China
[11]Africa Union-Africa Centres for Disease Control and Prevention
[12]Sanofi, UK

List of Figures

S1 Incidence comparison over time 5
S2 Mortality comparison over time 6
S3 WHO vs IHME incidence differences by HIV status 7
S4 Incidence and notifications for 30 countries 8
S5 Prevalence vs prevalence survey. 9
S6 Implied duration by HIV status 10
S7 Case Fatality Ratio vs Case Detection Ratio 11
S8 Implied duration by age and sex 12
S9 Implied duration over time 13

Preamble

The figures are generated with publicly available data from the World Health Organization (WHO) and Institute for Health Metrics and Evaluation's (IHME's) Global Burden of Disease Collaborative Network Global Burden of Disease Study 2019 (GBD 2019). They are available from:

- `https://www.who.int/teams/global-tuberculosis-programme/data`
- `https://vizhub.healthdata.org/gbd-results/`,

and are also included in the GitHub repository:

- `https://github.com/petedodd/ihmexplore`

for this article, which includes the R code for analysis & visualisation.

Some plots use ISO3 codes to refer to countries, which are provided on the next page in Table S1 for reference.

Panel membership

Prasada Rao Jvr (panel co-lead; former Special Envoy of UN Secretary General, India), Richard White (panel co-lead; LSHTM, UK), Faith Nekabari Nfii (Public Health Specialist, Africa Union-Africa Centres for Disease Control and Prevention), Pete Dodd (The University of Sheffield, UK), Michael Klag (Dean Emeritus, Johns Hopkins Bloomberg School of Public Health, US).

The panel was supported by the Secretariat of the Global Burden of Disease Independent Advisory Committee (GBD IAC) represented by Edmond Ng (Senior Statistical Analyst), and Nabila Shaikh (Research Assistant, LSHTM).

Expert group membership

Jing Wu (China CDC, China), Leigh Johnson (University of Cape Town, Sout Africa), Ibrahim Abubakar (University College London, UK), Nim Arinaminpathy (Imperial College, UK), Frank Cobelens (The Amsterdam Institute for Global Health and Development, Netherlands), David Dowdy (Johns Hopkins Bloomberg School of Public Health, US), Kathy Fiekert (KNCV, Netherlands), Philippe Glaziou (WHO, Switzerland), Alison Grant (LSHTM, UK), Rein Houben (LSHTM, UK), Joshua Salomon (Stanford University, US)

iso3	country name
AGO	Angola
BGD	Bangladesh
BRA	Brazil
KHM	Cambodia
CAF	Central African Republic
CHN	China
COG	Congo
PRK	Democratic People's Republic of Korea
COD	Democratic Republic of the Congo
ETH	Ethiopia
IND	India
IDN	Indonesia
KEN	Kenya
LSO	Lesotho
LBR	Liberia
MOZ	Mozambique
MMR	Myanmar
NAM	Namibia
NGA	Nigeria
PAK	Pakistan
PNG	Papua New Guinea
PHL	Philippines
RUS	Russian Federation
SLE	Sierra Leone
ZAF	South Africa
THA	Thailand
TZA	United Republic of Tanzania
VNM	Viet Nam
ZMB	Zambia
ZWE	Zimbabwe

Table S1: Key of ISO3 codes and country names

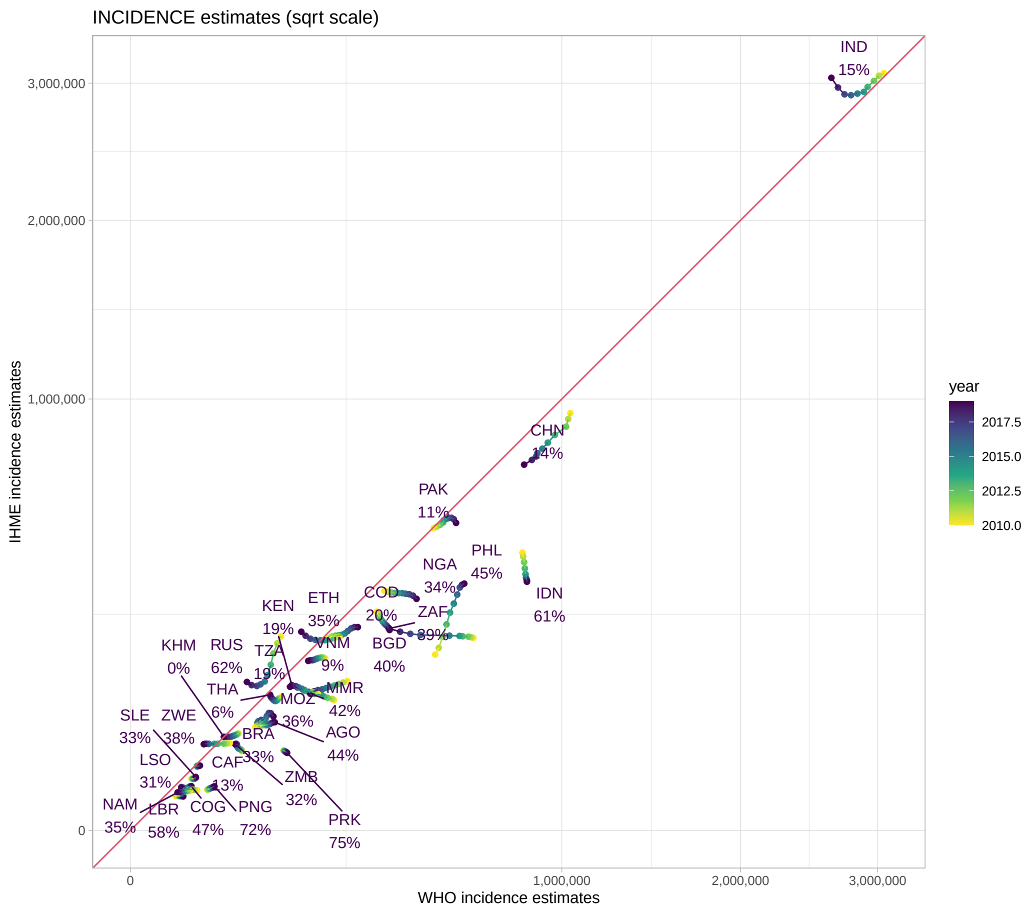

Figure S1: Tuberculosis incidence comparison over time. The red line represents equality. Percentages are the magnitudes of Institute for Health Metrics and Evaluation (IHME) estimate differences relative to World Health Organization (WHO) estimates in 2019. Country codes are shown in Table S1. sqrt = square root

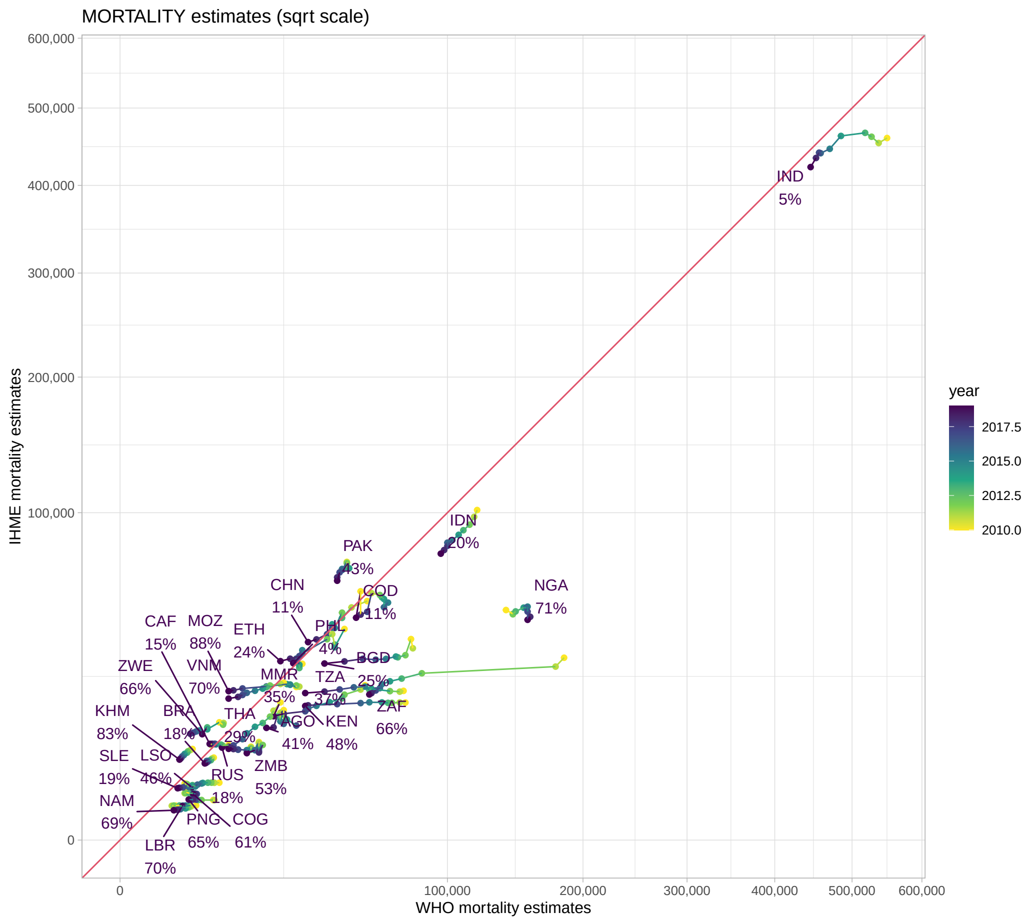

Figure S2: Tuberculosis mortality comparison over time. The red line represents equality.
Percentages are the magnitudes of Institute for Health Metrics and Evaluation (IHME) estimate differences relative to World Health Organization (WHO) in 2019. Country codes are shown in Table S1. sqrt = square root

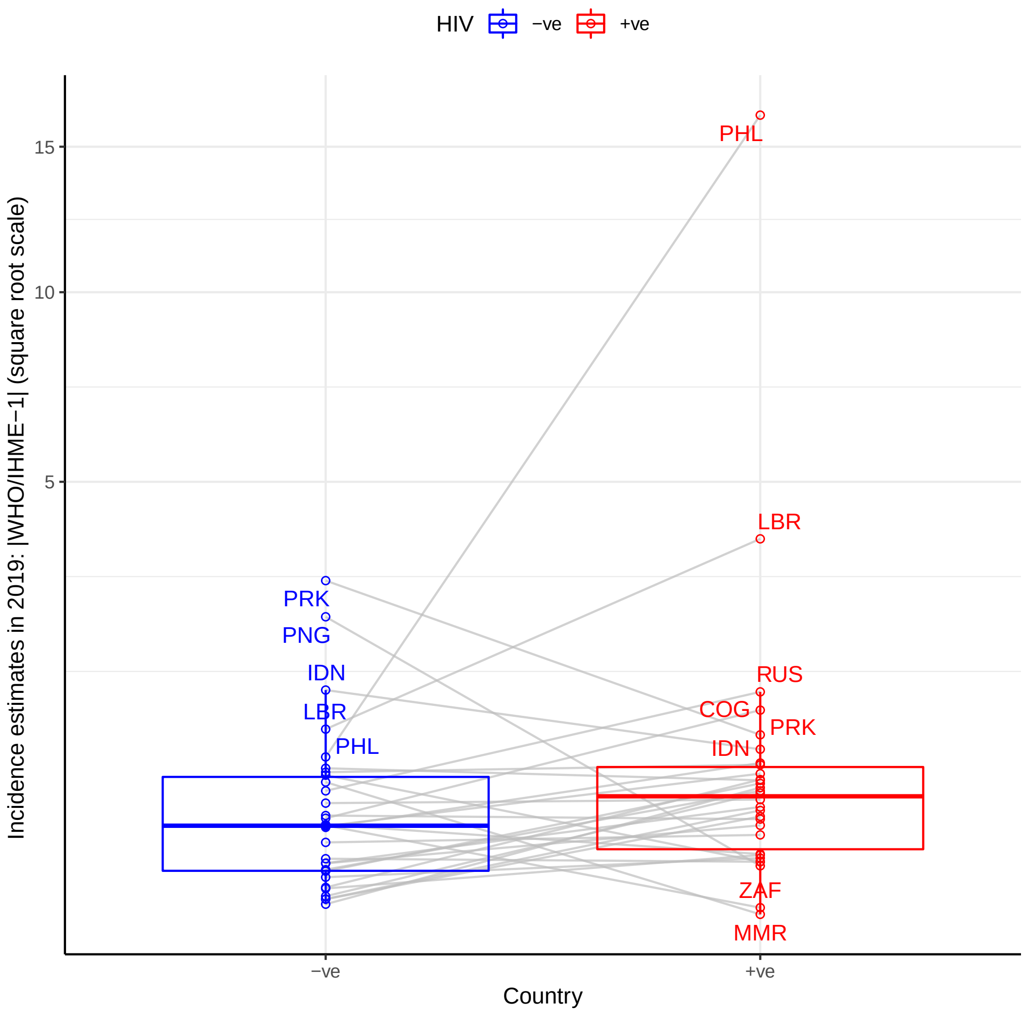

Figure S3: World Health Organization (WHO) vs Institute for Health Metrics and Evaluation (IHME) tuberculosis incidence differences by human immunodeficiency virus (HIV) status. The difference is the absolute difference as a fraction of the IHME estimate. Lines join country HIV± values. Some outlying countries are labelled. Country codes are shown in Table S1.

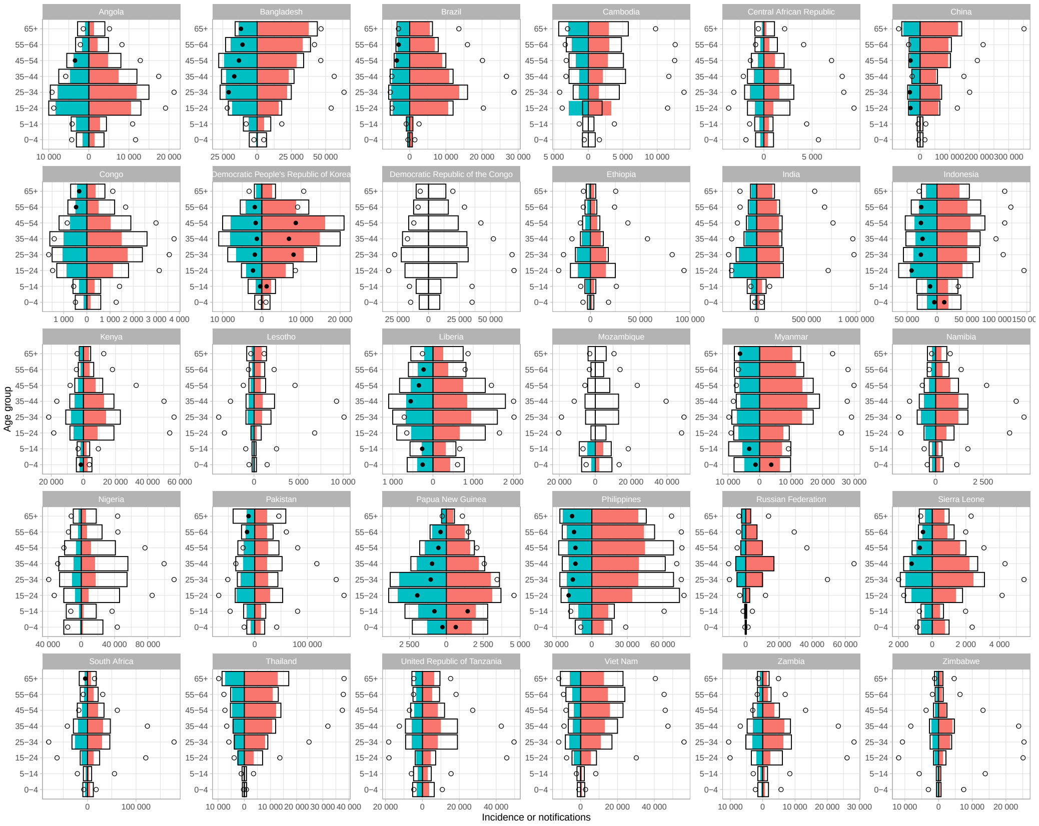

Figure S4: Tuberculosis incidence and notifications for 30 high tuberculosis burden countries in 2019. Coloured bars are notifications; open bars are World Health Organization incidence estimates; circles are Institute for Health Metrics and Evaluation Global Burden of Disease incidence estimates. Filled circles suggest a case detection ratio greater than one according to these estimates. Men to the right; women to the left.

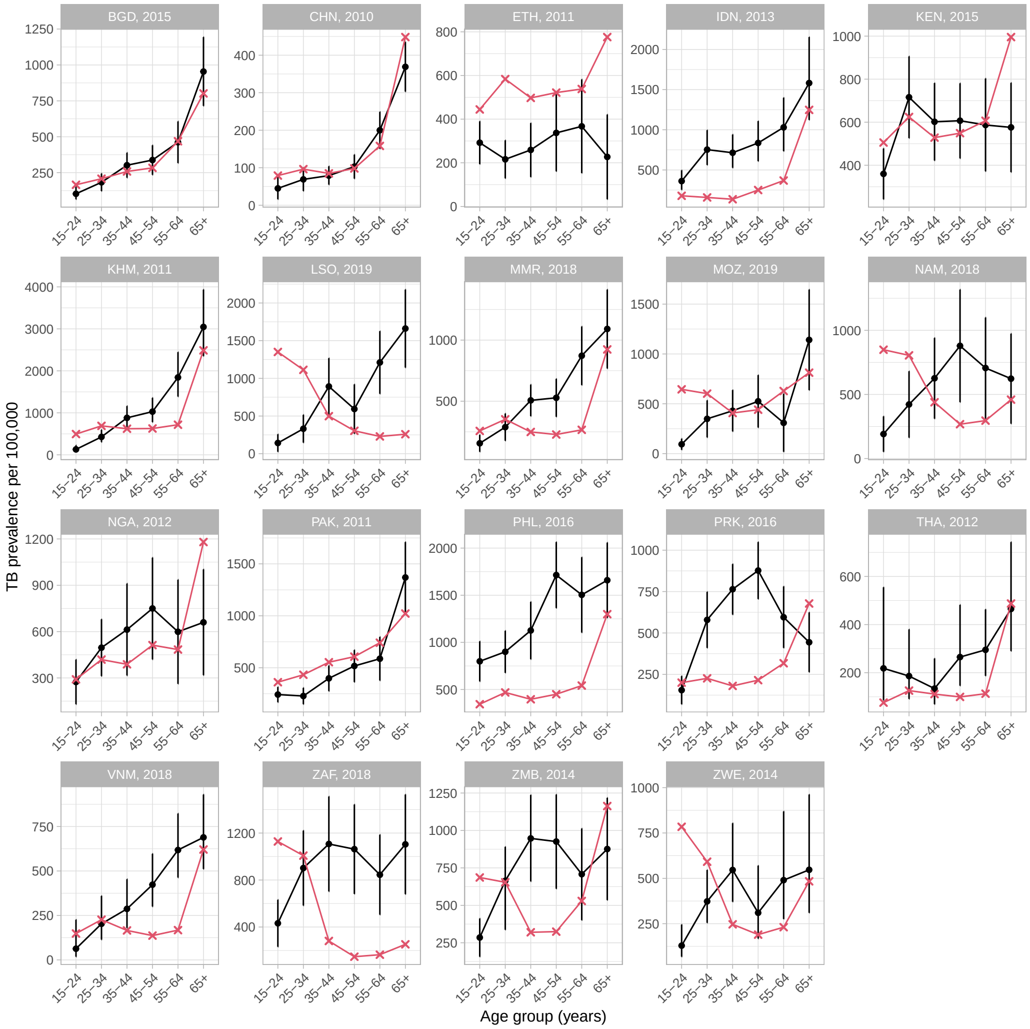

Figure S5: Tuberculosis prevalence vs nationally-representative prevalence survey. Red= Institute for Health Metrics and Evaluation all tuberculosis (TB) prevalence point estimate; black=prevalence survey bacteriologically-confirmed TB prevalence and 95% confidence interval. Bacteriologically-confirmed TB is a subset of all TB. Country codes are shown in Table S1.

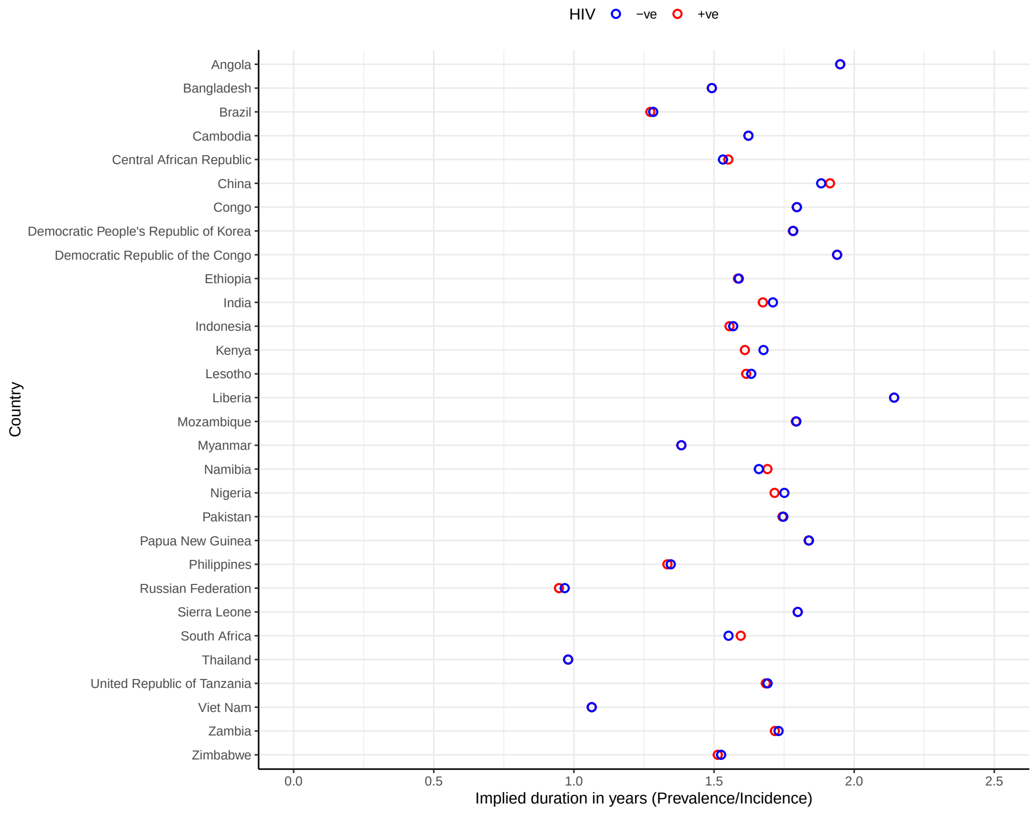

Figure S6: Implied tuberculosis duration by human immunodeficiency virus (HIV) status. Duration is calculated as estimated prevalence divided by estimated incidence. HIV-associated tuberculosis (TB) is thought to have shorter duration than TB disease in HIV uninfected people. Some red (HIV+ve) and blue (HIV-ve) points are not distinguishable due to overlap. +ve/-ve = positive/negative

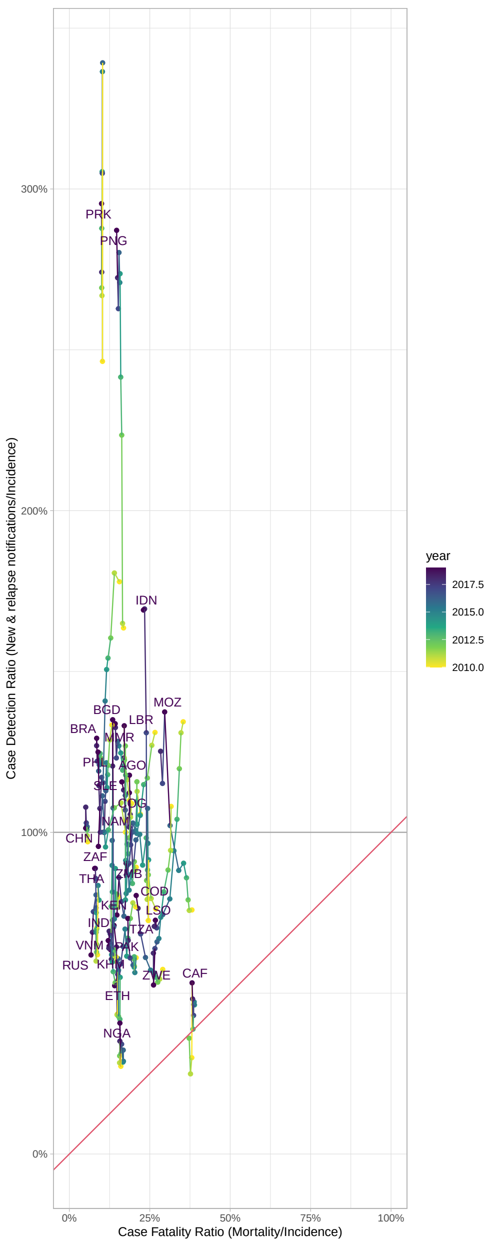

Figure S7: Institute for Health Metrics and Evaluation tuberculosis Case Fatality Ratio vs Case Detection Ratio 2010-2019. Case Fatality Ratio (CFR) is calculated as estimated mortality divided by estimated incidence. Case Detection Ratio (CDR) is calculated as notifications divided by estimated incidence. The red diagonal line is a reference line of equality. The horizontal grey line represents a CDR of 100%. CFRs are typically stable even for large changes in CDR. Country codes are shown in Table S1.

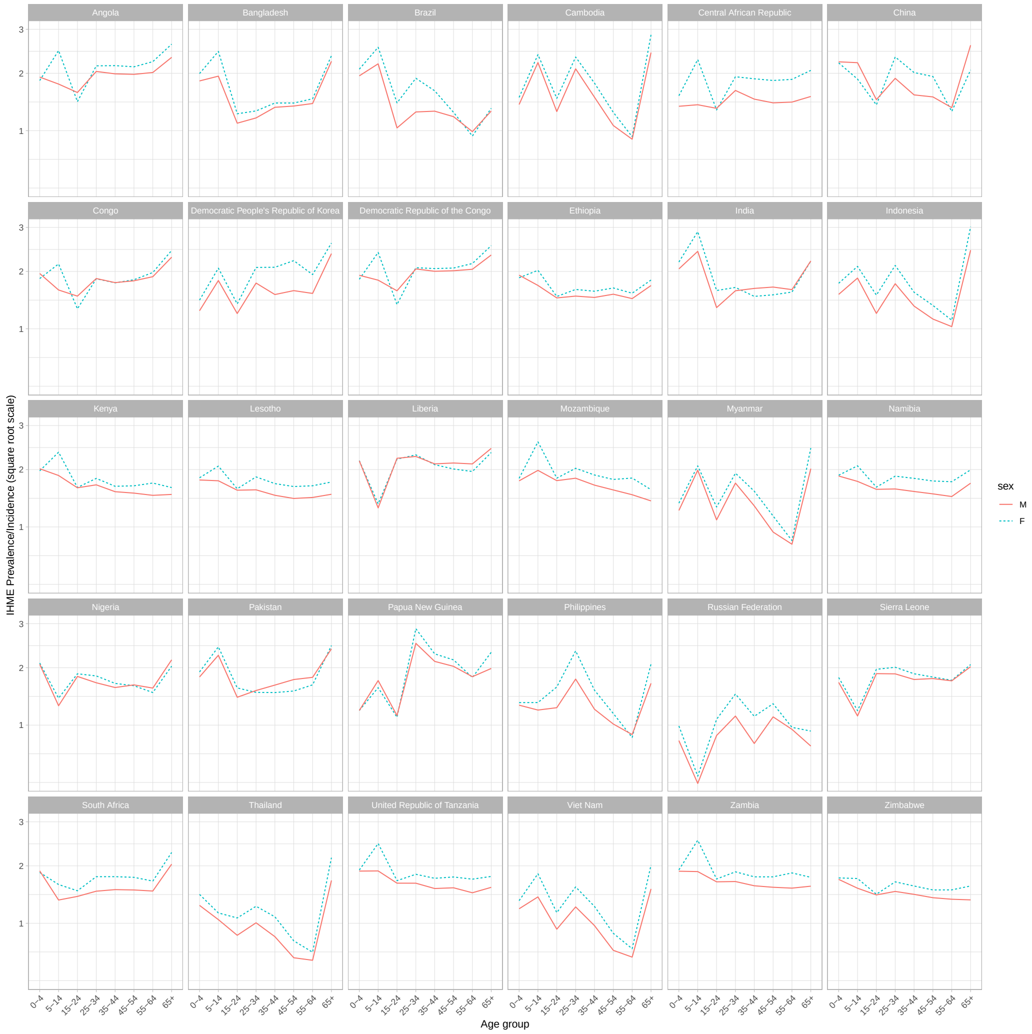

Figure S8: Implied tuberculosis duration by age and sex. Duration is calculated as estimated prevalence divided by estimated incidence. The y-axis is on a square root scale. Most settings have similar overall duration. Most settings have little difference in duration by sex. IHME = Institute for Health Metrics and Evaluation

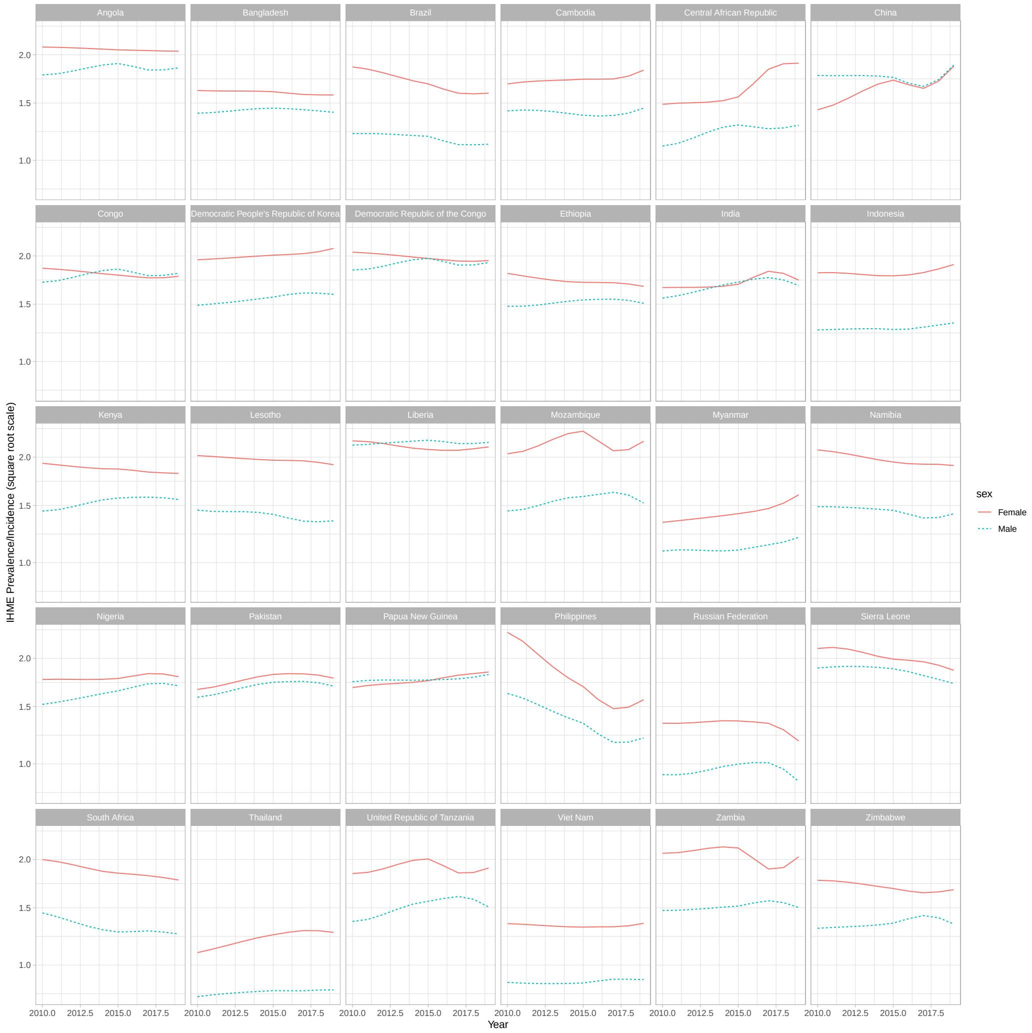

Figure S9: Implied tuberculosis duration over time. Duration is calculated as estimated prevalence divided by estimated incidence. Most settings show quite stable durations. IHME = Institute for Health Metrics and Evaluation
